# Identification of a new 130 bp *cis*-acting element in the *TsVP1 *promoter involved in the salt stress response from *Thellungiella halophila*

**DOI:** 10.1186/1471-2229-10-90

**Published:** 2010-05-18

**Authors:** Qinghua Sun, Feng Gao, Lei Zhao, Kunpeng Li, Juren Zhang

**Affiliations:** 1 School of Life Science, Shandong University, Jinan, China; 2 Department of plant science, University of Manitoba, Winnipeg, Manitoba, Canada

## Abstract

**Background:**

Salt stress is one of the major abiotic stresses affecting plant growth and productivity. Vacuolar H^+^-pyrophosphatase (H^+^-PPase) genes play an important role in salt stress tolerance in multiple species.

**Results:**

In this study, the promoter from the vacuolar H^+^-pyrophosphatase from *Thellungiella halophila *(*TsVP1*) was cloned and compared with the *AVP1 *promoter from *Arabidopsis thaliana*. Sequence analysis indicated that these two promoters had seven similar motifs at similar positions. To determine which tissues the two promoters are active in, transgenic plants were produced with expression of the GUS reporter gene under the control of one of the promoters. In transgenic *Arabidopsis *with the *TsVP1 *promoter, the GUS reporter gene had strong activity in almost all tissues except the seeds and the activity was induced in both shoots and roots, especially in the root tips, when treated with salt stress. Such induction was not found in transgenic *Arabidopsis *with the *AVP1 *promoter. By analyzing different 5' deletion mutants of the *TsVP1 *promoter, an 856 bp region (-2200 to -1344) was found to contain enhancer elements that increased gene expression levels. Two AAATGA motifs, which may be the key elements for the anther specific expression profile, in the deleted *TsVP1 *promoters (PT2 to PT6) were also identified. A 130 bp region (-667 to -538) was finally identified as the key sequence for the salt stress response by analyzing the different mutants both with and without salt stress. GUS transient assay in tobacco leaves suggested the 130 bp region was sufficient for the salt stress response. Bioinformatic analysis also revealed that there may be novel motifs in this region that are the key elements for the salt stress responsive activity of the *TsVP1 *promoter.

**Conclusions:**

The *TsVP1 *promoter had strong activity in almost all tissues except the seeds. In addition, its activity was induced by salt stress in leaves and roots, especially in root tips. A 130 bp region (-667 to -538) was identified as the key region for responding to salt stress.

## Background

Salt stress is one of the major abiotic stresses for plants in the world. High concentrations of sodium in soils are deleterious to the growth and development of non-halophytes. Global crop production is affected by salinity stress and this problem is becoming more and more serious [1,2]. Most of the major crops are salt-sensitive and irrigation-induced soil salinization causes the lost of large tracts of agricultural land [3]. It is important to study the salt-tolerance mechanisms to improve crop plants.

Arabidopsis (*Arabidopsis thaliana*) has been considered an excellent model system for determining molecular pathways in plants since its genome was sequenced in 2000. In the past ten years it has been used extensively to investigate salt tolerance in plants [4]. However, as a true glycophyte, it is difficult for Arabidopsis to survive at even moderate salinity (~100 mM NaCl). Studying only Arabidopsis could not provide enough information on salt-tolerant mechanisms. *Thellungiella halophila *is a salt-tolerant close relative of Arabidopsis that has been considered a model system for studying salt tolerance in plants [1,5]. Because of the high similarity between these two species at the cDNA level (>90% nucleotide identity in cDNA sequences), the resources of Arabidopsis such as gene and protein information can be used to study *T. halophila*. Furthermore, *T. halophila *shared several advantages with Arabidopsis. It has a small genome, a short life cycle, enough seed production and can easily be transformed [1,5]. Interestingly, these two species have obvious differences in stress-tolerance while sharing high similarity in cDNA sequences. This may due to differences in the gene regulatory regions between these two species [4]. Therefore, it is important to study the promoter sequences of *T. halophila *to discover how they differ from those of Arabidopsis.

Strong constitutive promoters, such as the CaMV 35S promoter and the maize ubiquitin gene promoter [6,7], have been widely used in transgenic plants to express foreign genes. However, it may be harmful to express a foreign gene constitutively in host plants. This may lead to sterility, delayed development, abnormal morphology, yield loss, changes in grain composition or transgene silencing [8-13]. To solve this problem, a strong tissue-specific or inducible promoter can be used to restrict gene expression to only the required tissue or at a specific time. Arabidopsis promoters *rd29A *and *rd29B *were found to response to multiple stresses including high salinity, drought, cold and ABA [14]. Then with the development of cDNA microarray technology and sequencing of the Arabidopsis genome, many stress inducible genes were identified in Arabidopsis [15-18]. In addition, a large number of tissue-specific or inducible genes and their promoters have been identified from other species [19-23]. However, there has been no report of a promoter that responds to salt stress particularly in the root tips.

Vacuolar H^+^-pyrophosphatase (H^+^-PPase) genes play an important role in abiotic-stress tolerance. Transgenic plants overexpressing the vacuolar H^+^-pyrophosphatase gene *AVP1 *from Arabidopsis are much more resistant to high concentrations of NaCl and to water deprivation than wild-type strains [24]. Gao *et al*. cloned a novel H^+^-PPase gene named *TsVP1 *from *Thellungiella halophila*. The heterologous expression of *TsVP1 *or *AVP1 *in yeast mutant *ena1 *partly restored its salt tolerance [25]. Heterologous expression of *TsVP1 *in tobacco also improved its salt tolerance [25]. Although they have a similar function in salt tolerance, these two genes had different expression pattern under salinity stress. When treated with 200 mM NaCl solution, *TsVP1 *expression increased 3-fold in the aerial roots and 5-fold in the roots during the first 16 h. But *AVP1 *expression was not changed [25]. Thus, there must be some differences between the promoter regions of these two homologous genes.

In this report we cloned and analyzed the promoter regions of the *TsVP1 *and *AVP1 *genes. In transgenic Arabidopsis, the GUS reporter gene driven by the *TsVP1 *promoter was obviously induced under salt stress, especially in the root tips. However, the GUS gene driven by the *AVP1 *promoter was not changed under salt stress. By analyzing the sequences and a series of deletion mutants, we identified a 130 bp region from the *TsVP1 *promoter which may be the key sequence for responding to salt stress.

## Results

### Cloning the promoters from *TsVP1 *and *AVP1*

A pair of primers, PTsVP-UP and PTsVP-DOWN was designed for primary screening of the *Thellungiella halophila *genomic library. The PCR products were DIG-labeled and then used for the subsequent in situ hybridization. A positive clone containing the upstream sequence of the *TsVP1 *gene was isolated and sequenced. The intergenic region between *TsVP1 *and the next upstream gene was 4467 bp and the fragment beginning at -2200 bp upstream of the *TsVP1 *translational initiation site was taken as the full-length promoter. The genomic sequences around gene *AVP1 *were obtained from TAIR. The intergenic region between *AVP1 *and the next upstream gene was 4507 bp and the sequence -2200 bp from the translational initiation site was considered to be the full-length promoter. This fragment was amplified from Arabidopsis genomic DNA and sequenced.

### Sequence bioinformatics analysis

The regulatory elements in these two promoters were analyzed using online software PLACE and Plantcare. In the *TsVP1 *promoter 21 kinds of putative *cis*-elements responding to heat, light, drought, MeJA, salicylic acid, ABA and stress induction were identified (see Additional file 1a). In the *AVP1 *promoter there were also 21 kinds of putative cis-elements responding to heat, light, gibberellin, MeJA, salicylic acid, ABA, cold and auxin stimulation (see Additional file 1b). Although these sequences shared about 75% similarity, these two promoters contained only seven similar regulatory elements at similar positions (see Additional file 1). Reported known high salinity inducible cis-elements were not found in these two promoters although *TsVP1 *gene expression was obviously induced under salt stress.

### Construction of promoter-reporter plasmids and plant transformation

**Figure 1:**
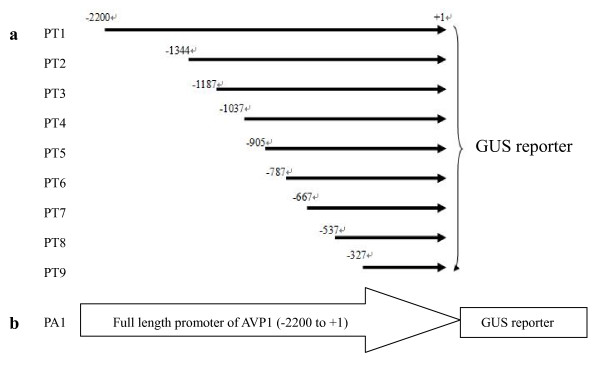
**The construction of the promoter-reporter plasmids**. a. A set of 5' deleted promoters fused to the GUS reporter gene. A series of 5' deletions of the TsVP1 promoter region were transcriptional linked to the GUS reporter gene. The number stands for the nucleotide position from the translational initiate site, ATG (A as +1). b. The full length AVP1 promoter was linked to the GUS reporter gene.

To compare the activities of these two promoters and determine the key sequences of the *TsVP1 *promoter, a set of reporter constructs were made by linking various lengths of the *TsVP1 *regulatory regions to the *uidA *reporter gene encoding β-glucuronidase (GUS) in the vector pCAMBIA1391z (Figure 1a). The full-length *AVP1 *promoter was also linked to the *uidA *reporter gene (Figure 1b). In addition to these constructs, vector pCAMBIA1304 was used as a positive control while vector pCAMBIA1391z was used as a negative control. These promoter constructs were analyzed using an Arabidopsis system since it has high transformation efficiency and there is a large amount of similarity between these two species. It was reported that cloned promoters usually retain their native expression patterns when transformed into related species [26]. In addition, Arabidopsis has been extensively used for promoter analysis from a wide variety of plant species.

Following transformation of these constructs into Arabidopsis, over 12 independent homozigous, single-copy transformants were obtained and analyzed using GUS histochemical staining and GUS enzymatic activity quantification for each construct.

### Characterization of the full-length *TsVP1 *promoter activity in Arabidopsis

**Figure 2:**
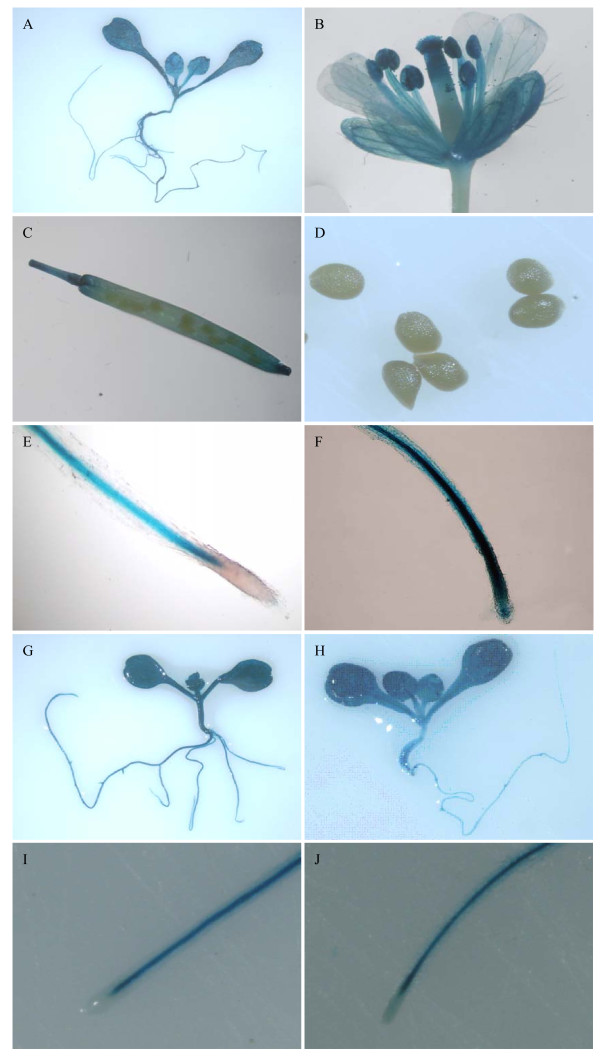
**Histochemical staining of transgenic Arabidopsis PT1 and PA1**. A. PT1 whole plant; B. PT1 flower; C. PT1 silique; D. PT1 seeds; E. PT1 root tip without salt stress. F. PT1 root tip with salt stress. G. PA1 whole plant without salt stress; H. PA1 whole plant with salt stress. I. PA1 root tip without salt stress. J. PA1 root tip with salt stress.

Histochemical staining of transgenic plants where GUS expression was driven by the full-length *TsVP1 *promoter yielded a whole-plant perspective of the promoter activity. GUS expression was present in leaves, roots, stems, flowers and silique pods, but not in the seeds (Figure 2). In 10-day old Arabidopsis plants, strong GUS expression was present throughout the whole plant (Figure 2A). In the flowers, GUS expression was also detected with an uneven distribution. GUS activity was much higher in the vascular tissue than in the other parts of the sepals and petals. In the stamens, GUS activity was more intense in the anthers than the filaments. In the pistils, GUS staining was detected mainly in the upper portion, especially in the stigma (Figure 2B). In the siliques, GUS expression was present in the pods but not in the inside seeds (Figure 2C, D).

**Figure 3:**
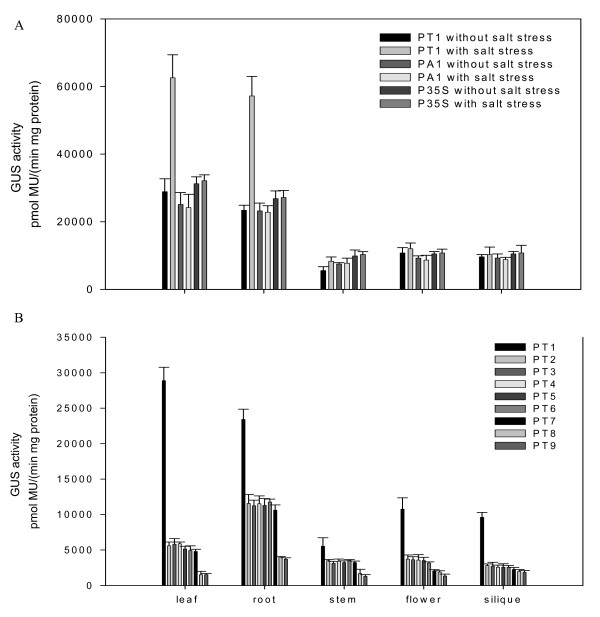
**GUS enzymatic activity quantification of different parts of transgenic Arabidopsis**. A. Expression of the GUS gene was driven by promoters PT1, PA1 and P35S under normal and salt stress conditions. B. Expression of the GUS gene was driven by promoters PT1 to PT9 under normal conditions.

After treatment with 200 mM NaCl for 16 hours, GUS activity in the 10-day old PT1 transgenic Arabidopsis plants was observably induced in the roots, especially in the root tips (Figure 2E, F). Because of the original high expression levels of this promoter in the leaves, it was difficult to see a change in expression levels in the leaves just by staining. To see the full-length *TsVP1 *promoter expression pattern and to find the exact GUS activity changes after salt stress, we measured GUS activity in different parts of the plants from transgenic lines PT1 under normal and salt stress conditions (Figure 3A). GUS activity was much higher in the roots and leaves than in other parts of the plants under normal conditions. With salt stress treatment, GUS activity in the roots and leaves was induced about threefold, while GUS activity was only slightly changed in the other parts of the plants (Figure 3A).

The transgenic lines PA1 in which the GUS gene was driven by the full-length *AVP1 *promoter was also analyzed as a comparison. They had strong GUS activity throughout the whole plants under normal conditions. This was similar to PT1. But after treatment with salt stress, no visible induction of PA1 was found by GUS staining (Figure 2G, H, I, J). This was different from PT1. For the positive and negative controls, strong GUS activity was found in the P35S transgenic lines while no GUS activity was found in 1391Z.

We also measured the GUS activity in different tissues in PA1 and P35S plants under normal and salt stress conditions in the adult plants (Figure 3A). The result indicated that GUS activity in PA1 and P35S was not induced by salt stress. Under normal conditions, PT1 and PA1 had similar levels of GUS expression to P35S. But after treatment with salt stress, PT1 had a higher level of GUS activity than PA1 and P35S in the leaves and roots (Figure 3A).

### Characterization of the activity from the 5' deleted promoter fragments in Arabidopsis

To identify the key regions of this promoter, we created eight 5' deletion mutants as shown in Figure 1. These constructs were introduced into Arabidopsis and a number of independent homozigous, single-copy transformants were obtained each construct.

**Figure 4:**
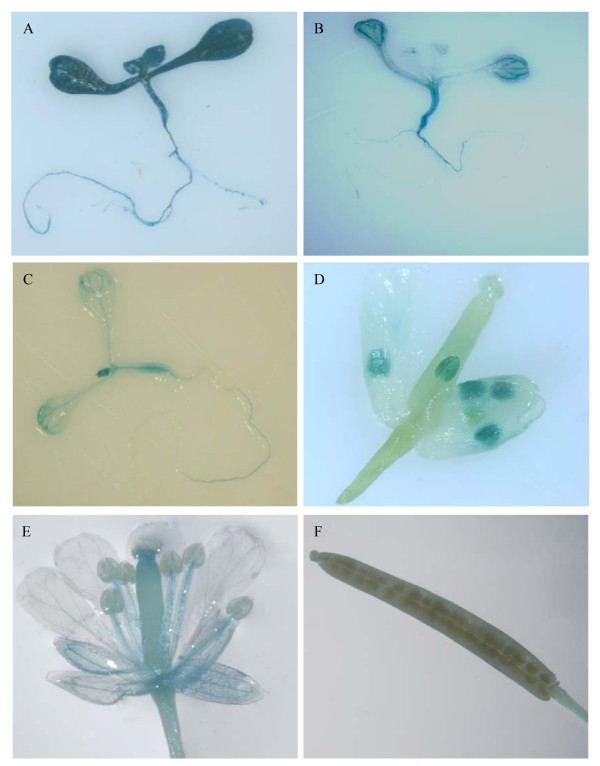
**Histochemical staining of different transgenic Arabidopsis lines where GUS expression was driven by different fragments of the *TsVP1 *promoter**. A. PT1 10-day old plant; B. PT2 10-day old plant; C. PT8 10-day old plant; D. a PT2 flower; E a PT7 flower; F. silique from a PT2 plant.

Transgenic line PT2 lacks the 856 bp region (-2200 to -1344), and had obviously reduced activity compared to PT1 (Figure 4A, B). In PT2 plants, GUS activity was detected mainly in the vascular tissue, especially in leaves (Figure 4B). In the flowers GUS staining was only found in the anthers (Figure 4D). For transgenic lines PT3 to PT6, a similar GUS expression pattern was detected both in the 10-day old plants and the adult plants. No obvious differences were found among these lines. PT7 had a similar expression level to PT6 except for the anther specific expression in flowers. In the floral tissue of transgenic line PT7, anther specific expression of GUS no longer existed. Instead plants of this line had weak GUS expression in the vascular parts of sepals and filaments. There was also weak GUS activity in the upper part of the pistil (Figure 4E). For PT8 and PT9, there was a similar expression pattern in the flowers compared with PT7, but weaker GUS expression in leaves and roots than PT7. PT8 and PT9 only had basal expression in the vascular tissue, both in the shoots and roots (Figure 4C). All of the constructs from PT2 to PT9 had a similar faint staining in the silique. In the petiole only very weak GUS expression was present. No visible staining was found in the other parts of the silique (Figure 4F).

We also measured the GUS activity in different tissues of PT1 to PT9 plants under normal conditions in the adult plants (Figure 3B). The result clearly indicated that PT2 plants had an obviously reduced activity in all tissues than PT1. For PT2 to PT7, They had a similar expression pattern except the flowers. PT7 had a reduced activity in flowere than PT6. PT8 and PT9 had a lower activity than PT7, especially in leaves and roots (Figure 3B).

### A 130 bp region was identified as the key region for responding to salt stress

**Figure 5:**
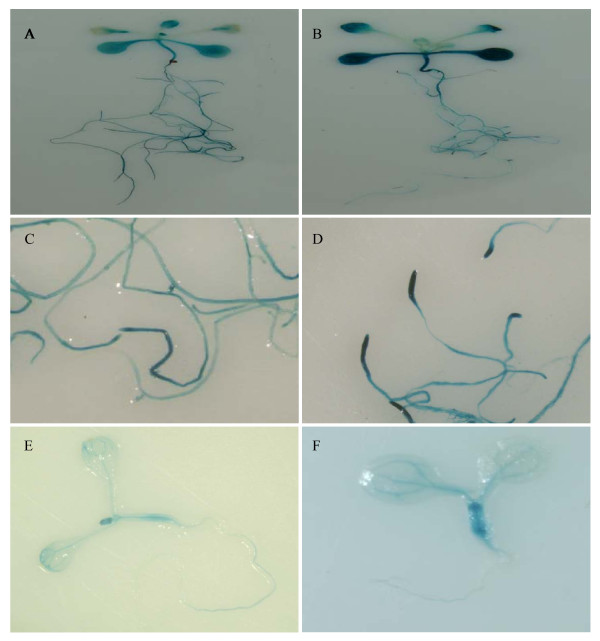
**Histochemical staining of transgenic Arabidopsis lines PT2 and PT8 under normal and salt stress conditions**. A. Whole PT2 plant under normal conditions; B. Whole PT2 plant under salt stress conditions; C. the roots from a PT2 plant under normal conditions; D. the roots from a PT2 plant under salt stress treatment; E. whole PT8 plant under normal conditions; F. whole PT8 plant under salt stress treatment.

To identify the specific region in the *TsVP1 *promoter involved in the salt stress response, all the 5' deleted constructs were analyzed in transgenic Arabidopsis by GUS staining both with and without salt stress. After treatment with 200 mM NaCl for 16 hours, transgenic line PT2 had induced GUS activity in both the leaves and roots (Figure 5A, B). GUS activity was strongly induced in the root tips (Figure 5C, D). This was the same as the PT1 transgenic lines where the GUS gene was driven by the full-length *TsVP1 *promoter (Figure 2E, F). In addition, because the GUS activity in PT2 was much weaker than PT1 in the leaves, the induction of GUS activity was visible in the PT2 leaves (Figure 5A, B). As mentioned above, PT2 and PT7 had similar levels of GUS activity under normal conditions. When treated with salt stress, these lines still had similar GUS expression patterns. GUS activity was obviously induced both in the shoots and roots in these transgenic lines, especially in the root tips (data not shown). But this induction disappeared in PT8 (Figure 5E, F), in which the GUS gene was driven by the promoter region from -537 to -1 relative to the translational initiate site. There was no salt stress induction of GUS activity found in PT9 either (data not shown). Comparing the promoter regions upstream from the GUS gene in the constructs PT7 and PT8, PT7 had 130 bp region (-667 to -538) that was deleted from PT8. This is the reason why GUS activity was induced by salt-stress in PT7 but not in PT8.

### The 130 bp region was sufficient for the salt stress response

**Figure 6:**
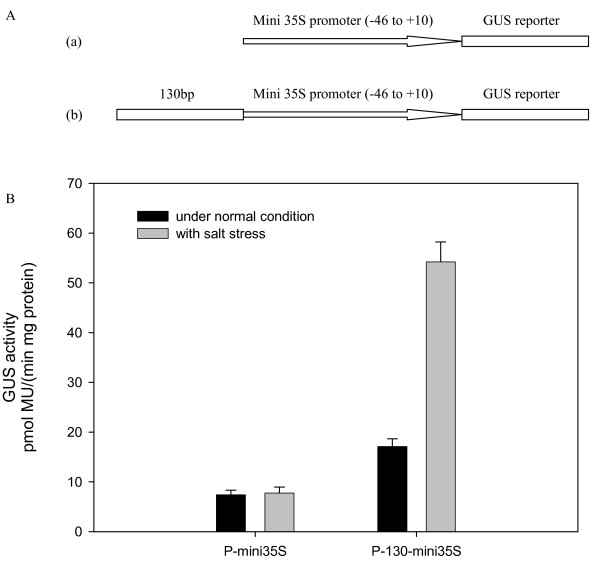
**GUS transient assay in tobacco leaves**. A. Fusion constructs used in the transient assay: (a), the control construct P-mini35S in which the GUS gene was driven by the mini 35S (-46 to +10) promoter. (b), the test construct P-130-mini35S in which the 130 bp region (-667 to -538) identified in the *TsVP1 *promoter was fused to the mini 35S promoter to drive the GUS reporter gene. B. GUS activities resulting from the transient transformation with constructs P-mini35S and P-130-mini35S under both normal and salt stress conditions.

To check if the 130 bp region (-667 to -538) identified in the *TsVP1 *promoter was sufficient for the salt stress response, we incorporated this 130 bp region with a minimal CaMV35S promoter (-46 to +10) to produce the promoter of P-130-mini35S and detected its activity under normal and salt stress conditions by *Agrobacterium*-mediated GUS transient assay in tobacco leaves (Figure 6). The P-130-mini35S had a near two-fold activity in comparison with the minimal CaMV35S promoter under normal condition. When treated by salt stress, the activity of the P-130-mini35S increased to about three-fold level, while the activity of the minimal CaMV35S promoter was not changed (Figure 6B).

GUS staining of the tobacco leaves was also carried out. The leaves infiltrated with the minimal CaMV35S*::gus *construct had only a very weak staining under both normal and salt stress conditions (see additional file 2A, B). The leaves infiltrated with the P-130-mini35S*::gus *construct had a higher GUS activity than P-mini35S under normal condition and its GUS activity was obviously induced by salt stress (see additional file 2C, D). These results indicated that the 130 bp region (-667 to -538) was sufficient for the salt stress response.

The constructs 1391Z and P35S were also used in this experiment as controls. The leaves infiltrated with 1391Z had no GUS activity while the leaves infiltrated with P35S had a very strong GUS activity (see additional file 2E, F).

### Bioinformatic analysis of the 130 bp region

**Figure 7:**
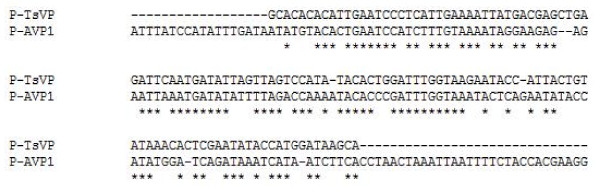
**Comparing of the identified 130 bp region in TsVP1 promoter to the corresponding sequence from the AVP1 promoter using the EBI tool ClustalW2**.

The 130 bp region (-667 to -538 relative to the translational initiate site) was analyzed using bioinformatics. First we searched for the known motifs present in this region with the online software PLNATCARE and PLACE. Four motifs including a G-box, a CGCTA-box, a box-III and a TC-rich element were found in this region (Table 1). No reported salt response motif was found in this region. In addition, although there was a different expression pattern under salt stress, the promoter sequences of the *TsVP1 *and *AVP1 *promoters had about 75% similarity. When we compared this sequence to the related sequence from the *AVP1 *promoter, a 127 bp region (-631 to -505) in the *AVP1 *promoter was found as the corresponding sequence to the identified 130 bp region in the *TsVP1 *promoter (Figure 7). Motif searching revealed that there was only one motif, a TCA-element functioning in the salicylic acid response in this 127 bp region in *AVP1 *promoter. Interestingly, none of the four motifs found in the 130 bp region from the *TsVP1 *promoter was present in the 127 bp region from the *AVP1 *promoter. This result again demonstrated that there were differences between these two promoters.

**Table 1:** *Cis*-elements existing in the 130bp region.

Cis-elements name	Sequence and position in the *TsVP1 *promoter	Putative function
G-box	-664 CAATTATTG -654	light response
CGCTA-box	-636 TGACG -632	MeJA response
box-III	-603 CATATACACT -594	protein binding site
TC-rich element	-586 GTAAGAATAC -576	defense and stress response

## Discussion

Previous studies have shown that Arabidopsis contains most, if not all of the salt tolerance genes one might find in halophytes. It was hypothesized that the large variations in salt tolerance or sensitivity among Arabidopsis and other halophytes was because of subtle differences in gene regulation [1]. In our previous work, it was discovered that *TsVP1 *and *AVP1 *have different expression patterns under salt stress, although they have similar functions in salt-tolerance both in yeast and tobacco [25]. To investigate the reason, we cloned and analyzed the promoters of these two genes. To our surprise, these two promoter sequences shared about 75% sequence similarity (data not shown). We then did bioinformatic analysis of these two promoters. As we expected, the *cis*-acting motifs present in these two promoters were not similar. They only shared seven identical regulatory elements at similar positions (see Additional file 1). Our results indicated that there were certain differences between these two genes in their regulation.

To study these two promoters in detail, we constructed promoter-reporter vectors and transformed Arabidopsis. All the transgenic plants were analyzed to understand the expression patterns of different promoter fragments. For the full-length *TsVP1 *promoter, the activity was as strong as the well known promoter CaMV 35S under normal conditions. After under salt stress, this promoter had even stronger activity in the roots and leaves (Figure 3A).

In Figure 3A we can see that PT1, PA1 and P35S all caused stronger activity in roots and leaves than in other parts of the plant. This indicated that PT1 and PA1 had full-length promoter activity and also suggested that the constitutive promoter CaMV 35S had different activity in different tissues. Some previous work also found that the widely used promoter CaMV 35S produced different levels of activity in different stages or different organs [27,28]. PT1 and PA1 had similar expression patterns in the absence of salt stress, but after salt stress, PT1 had obviously induced activity in the roots and leaves. This was consistent with our previous work [25].

Along with the development of plant genetic engineering, transgenic safety was also seriously considered. For most crops, the seeds are the final product that we use. Thus, it is important to protect the seeds from potential contaminating foreign genes. In our work we found that promoter PT1 had strong activity in almost every tissue except the seeds. This would be useful for its application in crop genetic engineering.

In the 5' deleted mutants, PT2 lacked a 856 bp region (-2200 to -1344), and had obviously reduced activity compared to PT1. We would expect one or more enhancers to be present in the -2200 to -1344 region. Bioinformatic analysis identified 16 putative cis-elements in this region (see Additional file 1). These elements included nine light responsive elements (an ACE, an AE-box, an AAAC-motif, a G-box, a box4, a box1, a GT1-motif and two SP1 elements), two protein binding sites (AT-rich element), a motif named HD-Zip1 associated with palisade mesophyll cells, an anaerobic induction responding element ARE, a drought responsive element MBS, a ABA responsive site ABRE and a heat stress responsive element HSE. No reported enhancer was detected in this region. Further study of this region is important to identify the minimum enhancer region.

In addition, PT2 showed an anther specific pattern of expression in the flowers. This expression pattern was also detected in deletion mutants PT3 to PT6 but not in PT7 to PT9. Mitsuda *et al*. had studied the *AVP1 *promoter in detail and also found its pollen specific profile [29]. They identified a 25 bp region within which there was an AAATGA sequence motif that had been identified in the regulatory region of the pollen-specific tobacco NTP303 gene [30]. In the *TsVP1 *promoter, two AAATGA sequence motifs were also found at the position -830 to -825 and -700 to -695. Both of these two positions was located in the promoter regions of deletion mutants PT2 to PT5, but only one AAATGA motif was present in the promoter region of PT6. There were no AAATGA motifs in PT7 to PT9. This result indicated that the AAATGA motif may be the key sequence for the anther specific expression pattern in flowers and only one AAATGA motif could drive the expression profile. There should be some other elements in the -2200 to -1344 region to drive the gene expression in other parts of the flower, so the PT1 plants did not have an anther specific expression profile in the flowers.

GUS expression was induced in both the leaves and the roots in PT1 under salt stress conditions. It was very interesting that the induction in the root tips was so intense (Figure 2E, F). Root tips are important parts of plants. They are the main regions for water and mineral uptake and gas exchange. Changing the gene expression levels in the root tips seems a meaningful way to engineer plants. Many stress inducible genes and their promoters have been identified [15-18]. In addition, some root specific promoters were also identified. Vijaybhaskar *et al*. identified a root-specific promoter from a glycosyltransferase gene of Arabidopsis [21]. Simon *et al*. identified a near root specific gene *FaRB7 *from strawberries and analyzed its promoter activity [31]. Schunmann *et al*. identified a promoter region controlling the root expression and phosphate deprivation response [20]. Kobayashi *et al*. identified two novel *cis*-acting elements, IDE1 and IDE2, from the barley *IDS2 *gene promoter, which confer iron-deficiency-inducible, root-specific expression in heterogeneous tobacco plants [19]. However, no promoter has been reported that was obviously induced in the root tips by salt stress. In this work we first report that the *TsVP1 *promoter can produce significant gene expression induction in the root tips by salt stress. This may supply new material for the genetic improvement of crops.

Analysis of the 5' deleted mutants under salt stress conditions revealed a 130 bp sequence (-667 to -538) in the *TsVP1 *promoter that may be the key region for responding to salinity. Agrobacterium-mediated GUS transient assay in tobacco leaves suggested that this 130 bp region was sufficient for the salt stress response. There were four cis-elements present in this region (Table 1). A G-box is a well-studied element that functions in the light response. The CGCTA-box was reported to be involved in MeJA-responsiveness. The Box-III was identified as a protein binding site and the TC-rich repeats may respond for defense and stress. It is possible that the TC-rich repeats may play a crucial role in this region because of their ability to respond for defense and stress [32]. This region may also contain a new element that is crucial for salt responding. Further research on this region, such as identifying the minimal key element in this region and the protein which plays an important role in the regulation of this promoter will be meaningful to reveal the salt-tolerant mechanism of *Thellungiella halophila*.

## Conclusions

In this study we cloned and analyzed the promoter of the salt inducible gene *TsVP1 *from *Thellungiella halophila*. It has as strong activity as the well-used promoter CaMV 35S in leaves, roots, stems and flowers. But no promoter activity was detected in the seeds. This was exciting for its application in crop engineering.

As we predicted, the activity of the *TsVP1 *promoter was obviously induced by salt stress while the *AVP1 *promoter was not. This indicated that there was difference in gene regulation between these two genes. And we also found that the induction of the *TsVP1 *promoter activity in roots was mainly in the root tips. Thinking of the important roles the root tips play, this result showed that it was meaningful to use the *TsVP1 *promoter in crop engineering. By analysis of different 5' deleted mutant, a 130 bp sequence (-667 to -538) of *TsVP1 *promoter was identified which may be the key region for its salt responsive ability. GUS transient analysis revealed that the 130 bp region was sufficient for the salt stress response. Bioinformatic analysis revealed that there may be novel motifs responding to salt stress exist in this region.

## Methods

### Isolation of promoter sequences

A genomic library from Thellungiella halophila was constructed following the protocols of the λBlueSTARTM *XhoI *Half-site Arms Kit plus PhageMaker^® ^KIT (Merck, Germany). To obtain the *TsVP1 *promoter sequence, a pair of primers, PTsVP-UP (5'-gtggcgtcggcgtttcttc-3') and PTsVP-DOWN (5'-cttggcgacgacactctgc-3'), were designed based on the cDNA sequence of *TsVP1 *and were used for the primary screening of this library. The DNA fragment amplified by PCR with these primers was then labeled using a DIG-High Prime kit (Roche Inc.) and the product was used for a secondary screening of the genomic library by phage in situ hybridization. Several clones that were positive for the *TsVP1 *promoter were isolated and sequenced. The orthologous gene of *TsVP1 *in Arabidopsis is *AVP1 *(AT1G15690). Its promoter fragment was obtained by PCR with a pair of primers named PAVP1-sense (5'-aataaatatctgacactgaact-3') and PAVP1-antisense (5'-cttctctcctccgtataagaga3') using Arabidopsis genomic DNA as template based on the public sequence from TAIR http://www.arabidopsis.org.

### Bioinformatic analysis of the promoter sequences

The upstream 2200 bp regions of these two genes were considered to contain the full length promoters. Regulatory elements in these regions were analyzed using the online program PLACE (a database of plant cis-acting regulatory DNA elements) and Plantcare (a database of plant cis-acting regulatory elements, enhancers and repressors). These two programs are available at http://www.dna.affrc.go.jp/PLACE/[33] and http://bioinformatics.psb.ugent.be/webtools/plantcare/html/[34], respectively.

### Construction of the promoter-reporter plasmids

Nine DNA fragments containing different 5'-deleted series of the *TsVP1 *promoter region [-2208, -1344, -1187, -1037, -905, -787, -667, -537 and -327 bp to -1, (the translation initiation site was designated as "+1")] were amplified by PCR with the appropriate forward (T1F, T2F, T3F, T4F, T5F, T6F, T7F, T8F, T9F) and reverse primers (PTsVP1R) that contained a *BamHI *site at the 5' end of each primer. The amplified products were sequenced by Biosune Company (Biosune, Beijing, China). Then these confirmed fragments were cloned into the *BamHI *site located before the *gus *reporter gene [35] in vector pCAMBIA1391Z (Cambia, Australia). The resulting plasmids were named PT1, PT2, PT3, PT4, PT5, PT6, PT7, PT8 and PT9, respectively.

The full-length *AVP1 *promoter was also amplified by PCR with primers PAF and PAR, sequenced and then cloned into the *BamHI *site in vector pCAMBIA1391Z. The recombined plasmid was named PA1. In addition to these constructs, a vector named pCAMBIA1304 containing the CaMV35S promoter upstream from the *uidA *reporter gene was used as a positive control, while the vector pCAMBIA1391z with no promoter upstream from the *uidA *reporter gene was used as a negative control.

The following primers were used:

T1F (5'-aatggatccttttccccaagatttgcta-3'), T2F (5'-attggatccattggagggttgcac-3'), T3F (5'-tacggatcctgtgcaccttatcttgtaca-3'), T4F (5'-aacggatcctcaagttgcgaaagtactgt-3'), T5F (5'-ttcggatccagatatcaatccgcacatg-3'), T6F (5'-tgtggatcctaatacacatggtttgttg-3'), T7F (5'-tgaggatccgcacacacattgaatccctc-3'), T8F (5'-ggaggatcctaagattcacctaattaatt-3'), T9F (5'-tgtggatcctgtctgataactatcggt-3'), PTsVP1R (5'-tggggatccctctcctccgaatagagaaa-3'), PAF (5'-aatggatccctgacactgaactaaatcga-3'), PAR (5'-tgaggatcccttctctcctccgtataaga-3').

### Plant culture and transformation

Arabidopsis thaliana ecotype Columbia (Col) were grown in a phytotron under the conditions of 22/16°C day/night temperatures, cool white light at a intensity of 8000 lux, 16 h/8 h light/dark cycle, 75% humidity. The recombinant plasmids were introduced into *A. tumefaciens *strain GV3101 and then transformed into Arabidopsis using the flower dipping method [36]. Transformants were selected by plating seeds on MS plates containing 20 mg/l hygromycin B. The positive transformants were confirmed by PCR using primers HPT-F (5'-cttctgcgggcgatttgtgt-3') and HPT-R (5'-ttcgatgtaggagggcgtgga-3') designed specifically for the hygromycin resistance gene in this vector. The homozygous transgenic plants were confirmed by genetic analysis of the segregation ratio of later generations. The copy number of transgenes was analyzed using the method described by Kihara T *et al*. [37].

### GUS histochemical and fluorometric analysis

GUS histochemical staining was performed using identified homozygous, single-copy transgenic plants and the method described originally by Jefferson [35] with a little modification. In brief, the plant tissues were incubated at 37°C overnight in a 100 mM sodium phosphate buffer (pH 7.0) containing 0.1% Triton X-100, 10 mM EDTA, 1 mM X-gluc and 0.5 mM potassium ferricyanide. The stained tissues were then washed several times with 70% ethanol to bleach the chlorophyll. Quantitative assays were performed with 4-MUG as the substrate. Plant tissues were harvested and homogenized in a 50 mM sodium phosphate lysis buffer (pH 7.0) containing 0.1% Triton X-100, 0.1% sodium lauroyl sarcisine, 10 mM EDTA and 10 mM β-mercaptoethanol. After centrifugation at 5000 × g for 10 min, the GUS activity was assayed using the resultant supernatant at 37°C in lysis buffer containing 1 mM 4-MUG. The reaction was terminated by adding 200 mM Na_2_CO_3 _to a final concentration of 160 mM. Fluorescence was quantified using a FLUOstar Galaxy multi-well plate reader with the excitation and the emission filters set at 365 nm and 455 nm, respectively. The protein concentration was determined as described by Bradford [38].

### Agrobacterium-mediated GUS transient assay in tobacco leaves

For construction of the reporter vectors, the minimal 35S (-46 to +10) promoter was amplified by PCR with the primers P-35s-up (aatggatccaagtctcaatagcccttt) and P-35S-down (tgagaattccgtattggctagagcagc). The amplified products were sequenced by Biosune Company (Biosune, Beijing, China) and then these confirmed fragments were cloned into the *BamHI-EcoRI *sites located before the *gus *reporter gene in vector pCAMBIA1391Z (Cambia, Australia). This construct was named P-mini35S. The 130 bp region of the *TsVP1 *promoter (-667 to -538) was also amplified by PCR using the primers P130-up (atcaagcttgcacacacattgaatccc) and P130-down (cctggatcctgcttat ccatggtatat) and then confirmed by sequencing. These fragments were then inserted into the upstream of the mimi-35S promoter in the P-mini35S construction by using sites for *Hind*III and *BamH*I, and named P-130-mini35S. These plasmids were then introduced into the *Agrobacterium tumefaciens *strain EHA 105. *Agrobacterium*-mediated transient assay was performed on the leaves of 6-week-old tobacco plants as described previously [39,40]. Briefly, *Agrobacterium *cultures were collected by centrifugation at 5000 g and resuspended in the transformation buffer (10 mm MES, pH 5.6, 10 mm MgSO4, 100 μm acetosyringone) to an OD_600 _of 0.8. The leaves of 6-week-old tobacco plants were used for infiltration. The surface of leaves was slightly wounded before infiltration to increase the transformation efficiency. The infiltrated materials were maintained at 22°C in dark for 2 days. For salt stress the materials were treated with 200 mM NaCl for 16 h. Finally the infiltrated leaves of each construct both with and without salt-stress were collected for GUS activity measurement.

## Abbreviations

4-MUG: 4-methylumbelliferyl-β-glucuronide; ABA: Abscisic Acid; CaMV: cauliflower mosaic virus; cDNA: complementary DNA; EDTA: ethylene diamine tetraacetic acid; GUS: β-glucuronidase; MeJA: methyl jasmonate; MS Medium: Murashige and Skoog Basal Medium; PCR: Polymerase chain reaction; X-Gluc: 5-chloro-4-bromo-3-indolyl-glucuronide.

## Authors' contributions

QS and FG cloned and analyzed the two promoters. QS and LZ did the construction of promoter-reporter plasmids, plant transformation, transgenic Arabidopsis analysis and *Agrobacterium*-mediated GUS transient assay in tobacco leaves. KL and JZ contributed to the design of experiments. QS wrote the manuscript. All authors read and approved the final manuscript.

## Supplementary Material

Additional file 1 **(*Cis*-elements analysis of the promoter sequences of the *TsVP1 *and *AVP1*) 27K**. *Cis*-elements analysis of the promoter sequences of the *TsVP1 *(a) and *AVP1 *(b). The words in red were the putative elements sequence and the words below were the description of corresponding elements. The description of the seven *cis*-elements existing both in the *TsVP *1and *AVP1 *promoters with similar position were set in blue. Click here for file

Additional file 2 **(GUS staining of tobacco leaves in the *Agrobacterium*-mediated transient assay with different constructs) 60K**. GUS staining of tobacco leaves in the *Agrobacterium*-mediated transient assay with different constructs. A. P-mini35S under normal condition; B. P-mini35S under salt stress condition;C. P-130-mini35S under normal condition; D. P-130-mini35S under salt stress condition; E. 1391Z under normal condition; F. P35S under normal condition. Click here for file
