# Supplementary material for: Identification of a new 130 bp cis-acting element in the TsVP1 promoter involved in the salt stress response from Thellungiella halophila
Source: BMC Plant Biol. 2010 May 18;10:90. doi: 10.1186/1471-2229-10-90 (PMC3017807; doi:10.1186/1471-2229-10-90)
Supplement: Additional file 1 — (Cis-elements analysis of the promoter sequences of the TsVP1 and AVP1) 27K. Cis-elements analysis of the promoter sequences of the TsVP1 (a) and AVP1 (b). The words in red were the putative elements sequence and the words below were the description of corresponding elements. The description of the seven cis-elements existing both in the TsVP 1and AVP1 promoters with similar position were set in blue. [file 1471-2229-10-90-S1.PDF]

## a, Bioinformatic Analysis of the *TsVP1* Promoter

```
-2200          AA GATTTGCTAC CTCCCATTAC GCCAAAAATA ATTTTAAAAA ATCATCTAGA
                            Sp1
-2148   TATTATACGT TTTCTAGATA CCAATAAAGT ATCTGACATG AATAAAAGAA TAAATAAAGG
           ACE
-2088   ACCACATGTT CTTAAAAATA TAACTCACAT CAATAAATCA AATAATTCAA CATTTTACCT

-2028   TTAAACATAG AAATGAACCA TCCGTGACTT TTATCAAAAT AAATATATTT CTATGAAGAA
          AT-rich element
-1968   CAATAGTTCG TCCATATATT TCAAGTTTCT ACGAATCTTA TTAAGATAGA TTGTACTGGT
                                 AE-box
-1908   CACCGTTTTA TGGCATTTTG TCAATATATA AAAGATTATA AACATAGAGG TATCACCAAA

-1848   TTTTGGTGAG GCTTTGATTG CCTTAATTAG AAAACAATAT AATTACGACA GCGACGATCA
             AAAC-motif
-1788   TCTAATCATG TCACGGAAAG TCGGAATTGT TACCGTGTAA AATCATCCGC TTCTTTCTTA

-1728   ATTCAATTAT TGTTACTTTA ATTAGTTGGA TTGGTTTGAA TTCTACGTTT ACACCTCTGC
          HD-Zip 1                        ARE
-1668   ATTTGGATAA CCTATGCCAT ATAGTATTGA TTATATAACT GATAACTAAG GGAACTAAAA
                                              MBS
-1608   TACTAAATCT TGACTGAAAG ATAATAAGAG AAGTCTCTAG TCCCTAATGT CAGCCACATA
      AT-rich sequence
-1548   CCTAGGAAAG ATTTTTTAGT TTTCATTACG TGGTAAATTT TCCAGATTGA TTAGCAACCT
                                  ABRE G-BOX
-1488   GCAAAAAAGG GAACAAGAGG GAGGAAGATA AAGCATGAGT GGTCACTACT CACTCACTGA
                            Sp1
-1428   TACTACAAGA AATTAATTAA TGCTAACAAA AATCCACAAT ATAAATTTTT AATTAAGATA
                     Box 4            GT1-motif      HSE
-1368   TTTTTTCAAA AGAGTATTTA TCCGATTGGA GGGTTGCACA CCATTCAATA CACATTAATA
          Box I                                                Box 4
-1308   CGTCACAGCC GCTCGAAAAG GCCTCCGCTA TATATCCATC TTTTTGCTAA AGAATTTTTT
      CGTCA-motif                          TCA-element          HSE
-1248   TTTTCGAAGA ATCATCAATT TACTTTATTC TTGGTCTGAG GAAATATCTA TATACGTGAA
                                                              G-BOX
-1188   GTGTGCACCT TATCTTGTAC AGGGCTTCCT TCGGAAAAAA AAAAAATCTT GTACAGGGCT
       chs-CMA2c
-1128   TCCCTCGTAT GAAGAAGAGA TAAACTCGTA AAAAGGTTTT GGAATCCAAC TTAATGGAAC

-1068   AAATGCCAAA ATAATTTAGT ACAACGTCGA ATCAAGTTGC GAAAGTACTG TAGAATCGAT

-1008   TCCTATACAG AGACGAAACG AGTTCTTATT GCACTTTATA ATATTGATTA AAAACTGAAA
            5UTR Py-rich stretch
-948   CTTTTTTTGT TTCTCCGAAT ATTGTTATCT TTATTTCACA GCTAGATATC AATCCGCACA
       HSE
-888   TGCATATAAT TAGTTTATCT TTTCACAAAA AAGTATATCC CCAAATAAAA TCCCACGTAA
                                                         ABRE G-BOX
-828    ATGAAAACAC AGTTTTAATT TTTTTTTCAT AGTGTTTACG ATAATACACA TGGTTTGTTG
                                                            ARE
-768    TAATTAAAAA GATAGATACG GTTTTTTTTC AACTGAAAAT TTGACTTTGT ATTTATCCTA
          TCA-element
-708    ATGATATCAA ATGATTTAAC CATATCCATA TCTGATAGTA TGCACACACA TTGAATCCCT
```

GT1-motif G-BOX

-648 CATTGAAAAT TA**TGACG**AGC TGAGATTCAA TGATATTAGT TAGTC**CATAT ACACT**GGATT
CGTCA-motif Box III
-588 TG**GTAAGAAT AC**CATTACTG TATAAACACT CGAATATACC ATGGATAAGC ATAAGATTCA
TC-rich repeats
-528 CCTA**ATTAAT TAAT**TTCCAC CAGTGAGAAA AAAAGGAAAA CTA**ATTAAT**T TCTA**CACGTA**
Box 4 Box 4 G-BOX
-468 GACAATTGAC TACGAATCAA AAAGAGCGCT AATCGTGTAC CTGACAGTC**G GACGG**ACACA
A-box
-408 AGCGCATGGA TGCA**CACGTA** GTTGGTGTCT CTTACCCTAA AATCGACGGT GAAGATG**GAC**
G-BOX
-348 **ACGAGCG**TTA CTTGTCCCTC GTGTCTGATA ACTATCGGTA GACGAAACGA GATTATACCT
ABRE
-288 TCCGA**CAAT**T TGGAGCGTCG GCTGCACGCG CTTTCTAATC AAAAAAAAGA AAAAAAAAGA
CAAT-BOX
-228 AACGAGGGGT GTAGCGTAAA GAACGGTGTA ACTAACCGTA CCAGATTCCA ACTTTCGTTG

-168 TGGATGTGAG CATCCAAGAG GAGGAGAGTC AGTG**TTATAA A**ACGACACGA TATCCTCACC
TATA-BOX
-108 GAGTTTACGC CTTCATTTCA TCATCTCGTC GAAAACACTT CCCTTCCTTT CTCTCTACTC

-48 TCTCTCTCTC GTTATCTTCG GTTTCTGCTT TCTCTATTCG GAGGAGAGAT G

## b, Bioinformatic Analysis of the *AVP1* Promoter

-2200 CTGACACTGA ACTAAATCGA TAAAAAGTAT AAATAAAGCA CCATATGTTA TTAAAATATA

-2140 ATTCACAT**AA AAAATATC**AA ATAATTCATA GATTTGCCTA TAAGCAA**ATG AC**TCGTCCGT
HSE Skn-1_motif
-1080 GACTGTTAAA TGAA**ATTAAT** CGGTACAAG**T TATCTAT**GGA AAACAGTTCA ACTATTTAAC
Box 4 GA-motif
-2020 TATATCTTCT ACTACAGTGT CTTTTTCGTT CTAATTGAGT AATTCA**TAAA CGTTTA**ACTA
ACE
-1960 ATTCGATTGA TACTCATCAA **CGCCTTATAG** CATTGTCAAT ATACAACTAT AAACATTGAG
I-BOX
-1900 ATGTCACCAA ATTTTGGTGA GGCTAAATAG AAAACATTAT AATTACGACG ACGACGAT**AA**

-1840 **TCTAATCA**TA TATCATGGAA AGTCACAAA**T CTTAC**CGTGT AAAATCATTC GCCTCTTTCT
ATCT-motif TCT-motif
-1780 AATT**CAATTA TTGTTTT**AAC TTTAATTAGG TGGAT**TGGTT T**GAATTCTAC GTTTACCACA
HD-Zip 1 ARE
-1720 CCAGTCACCA CTTGGATAAA CCTTTAGGCC ATGAATGATA TTATATAATT TA**GAAAGAA**A
AAGAA-motif
-1660 TGGAAACTAA ATCGTGATTG AAAGATAAAA GTATGTCAGC CACAAAACTA GGAAAGATTT

-1600 CCTTTACATG ATAATAATTT TTTCGAGATT CATTAGCAAC CTG**CAAAAGG G**AACAAAGAT
P-box
-1540 AAAGCATGAG TGGTCACTAG TACTCACTGA GTACCCAAAA AAAACACAAT TAAATGCTA**A**

-1480 **GAAAATTCC**A CAAAATAATT TTTT**GGTTAA** GATATTTTAT TTTATATATA AAAGGTATT
HSE GT1-motif
-1420 TATCCGATTG GAGGGTTGCA CACCATTCAA TACACTTAAT TA**CGTCA**GCC GTTCGAAAAG
CGTCA-motif

-1360 GCCCTCCGAT ATATCCTCCT CTCTCACAAA CTTCTTCAAT TTTGTATTCA CTGGCAATAT

-1300 CTATGTACGG TTTGGTGCAC TGTATCTTCT TA*CACGAC*TG TCTCGTATCG TATGAAG*ATG*

G-BOX

-1240 *AGATTAA*AAT GAAAAAGTAT TGG*AATCTAA CCT*AATCGAA CAAATGCCAA AAATAATTTA

ATCT-motif ATCT-motif

-1180 GTACAACGTC GAATCGAATT TGTGAAAGTA CTATAGAATC GATTCACATT A*GAGACGAAT*

TCA-element

-1120 *A*CGAGTTTTT ATTCCACTTT AAATTTCATC GTAAACTGAA AAGTTTTTTT TTTTTTTTTT

-1060 TGCTTTGCAT TTTTCTTTTT TGCTTTATCT *GACACGTA*GA T*ATTAAT*CAG CACACGGATG

G-BOX Box 4

-1000 TAGTTTTATC TCTTTGCCAA AAAA*AGAGAG T*CAAATCCTC A*AATTTAATC C*TACATAAAT

GAG-motif ATCT-motif

-940 GAGAATTTTG TTATTGTTAA TCATAGCTTT TACAATTATA GCAAGCAT*TT ACTTTACC*CC

AAGAA-motif

-880 AATAAAGTTT ATGGCAAGCA TTTATATATC GTGGATTGGT AAATTAACAA TTTTATACAG

-820 ATGGCCTGTT TTAATTAAA*G GGTTAGATT*T TTCACTATGG CTTTCATTTA AACAATTAAA

ATCT-motif

-760 AACCCTGATA TTTTAACTAC GTATTTCCAT TTATCCTAAT CATGAA*ATTA AT*ACAAAATG

Box 4

-700 ATTTAGGCTC TTAACTATAT CTTCTTTTTT TATTGTTCAA AGCTCTTTAC TATTTATCCA

-640 TATTTGATAA TATGTACACT GAAT*CCATCT TTGT*AAAATA GGAAGAGAGA ATTAAATGAT

TCA-element

-580 ATATTTTAGA CCAAAATACA CCCGATTTGG TAAATACTCA GAATATACCA TATGGATCAG

-520 ATAAATCATA ATCTTCACCT AACTAA*ATTA AT*TTTCTACC ACGAAGGGGG ATTGACTACA

Box 4

-460 AAAC*CCGAAA* AGTACTAATC ATGTACTTCC AGATCCGACG GCTAAAAACG CATCGATCAA

LTR

-400 *CACGT*AGTAG GTGTCTTTCG CCCTAAACTC *AACGAC*CAAG ATGAACTCAA GCTTTACTTT

G-BOX TGA-element

-340 GCT*GTCGTG*T CCGACAACTA CCGGATAGAT GAGGATGAAT CTAAATTCGG A*CAAAT*AGAG

G-BOX CAAT-BOX

-280 CGTAGTCAAC ACGCGCGTAA ATGAGTTCTG ATGGTGTAGC GTAAAAACCG TGTACCCAAC

-220 CGTACCGGAT TCCATCTATC TTTCTGGATG GGAGCATCCA AAGAGAAGAA GGAGAGAGCT

-160 AGTCT*TATAT AA*CGACACGA TATCCTCACC TATTTACGCC TCATTTTCTT CTTCACCTCT

TATA-BOX

-100 CTCTCTGTGT GTGTTCTGTG TTCTCTCTCT CGCGCGAAGC GGTTCTCTTT CTTTTGTTTA

-40 TTTGTTTTTA TTTGTTTTTC TCTTATACGG AGGAGAGAAG ATG
