# Supplementary figures and images for: Identification of a new 130 bp cis-acting element in the TsVP1 promoter involved in the salt stress response from Thellungiella halophila
Source: BMC Plant Biol. 2010 May 18;10:90. doi: 10.1186/1471-2229-10-90 (PMC3017807; doi:10.1186/1471-2229-10-90)

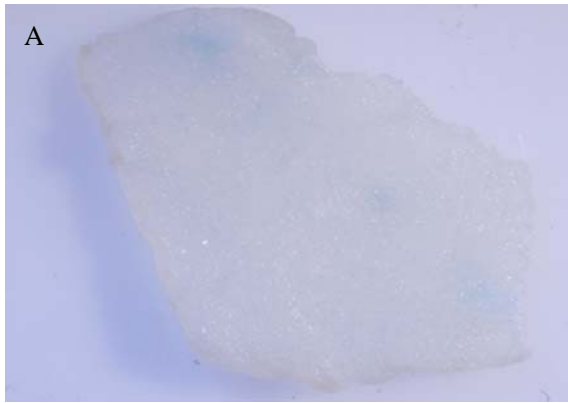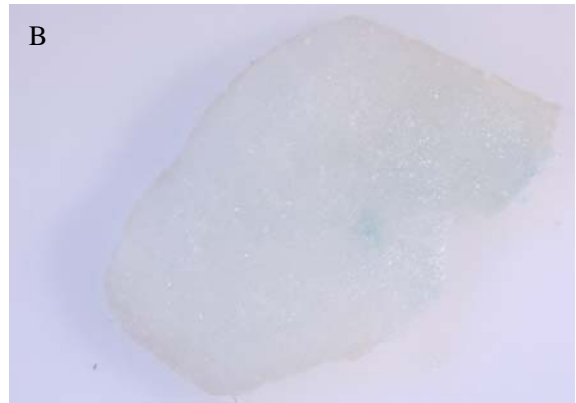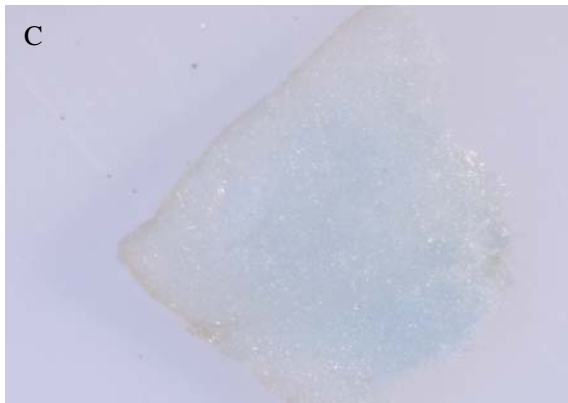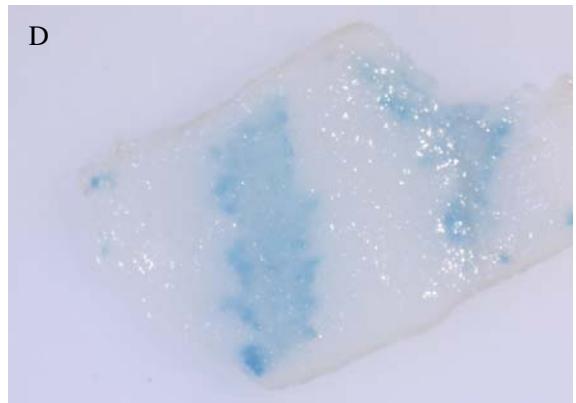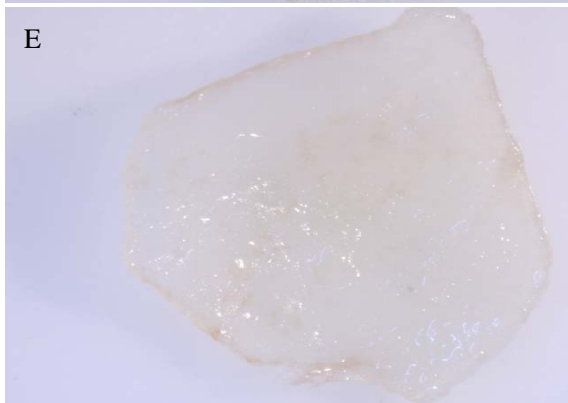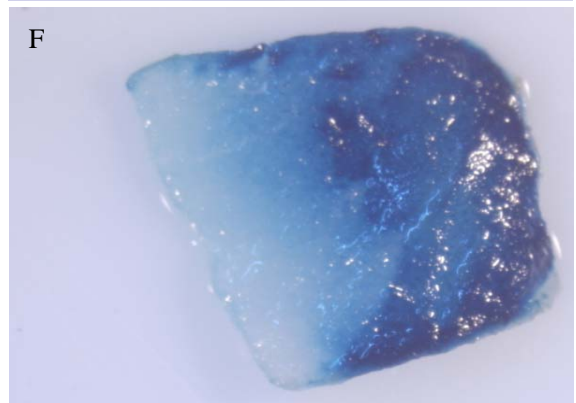

Supplement: Additional file 2 — (GUS staining of tobacco leaves in the Agrobacterium-mediated transient assay with different constructs) 60K. GUS staining of tobacco leaves in the Agrobacterium-mediated transient assay with different constructs. A. P-mini35S under normal condition; B. P-mini35S under salt stress condition;C. P-130-mini35S under normal condition; D. P-130-mini35S under salt stress condition; E. 1391Z under normal condition; F. P35S under normal condition. [file 1471-2229-10-90-S2.PDF]
